# Notes and Miscellany

**Published:** 1885-01

**Authors:** 


### NOTES AND MISCELLANY

=In Krupp's great gun manufactory, at Essen, compressed carbonic acid is used for the manufacture of what ice and seltzer water may be required by the workmen.

=Joy is heightened by exultant strains of music, but grief is eased only by low ones. "A sweet, sad measure" is the balm of a wounded spirit. Music lightens toil. The sailor pulls more cheerfully for his song.

=When John G. Whittier, the poet, was nineteen years of age, he left his first poem under the door of the office of the Newburyport (Mass.) *Free Press.* Week after week passed, and the young poet's heart sank within him, because he supposed his verses had been condemned. One day, while he was repairing a stone fence, the postman brought him a paper, and in the very first column was his poem. He was nearly wild with joy, and read it over a hundred times. Mr. Whittier says that no literary work in after life gave him such keen pleasure as his first poem.

="All paths lead to the grave." Such is the cynic's criticism of the allopath, the hydropath and the homœopath.

=The quantity of distilled spirits in the United States in October last was 115,949,235 gallons, of which the United States were taking care of 74,582,117 gallons in bond until the owners could find it convenient to pay the tax on it. The amount of human misery, the murders, the fires, the suicides, the defalcations, the loss of property and health, the divorces, the family shame and sorrow, stored up in this amount of liquor is simply incalculable.

=Relief may be secured from nervous neuralgia and sick headache by the use of Tongaline, which is not a cure-all, but which is a cure for nervous headache, neuralgia, rheumatism, gout and sciatica. No proprietary medicine has ever obtained such a strong endorsement from the medical profession.—*Medical Brief.* O. D. Norton, M. D., of Cincinnati, O., says of it : " Have used Tongaline in cases of neuralgic headache with success in almost every instance. In strictly neuralgic forms it is unexcelled."

="Now, there is Bunker Hill!" argued a Boston young lady, loftily; "a grand historic spot! What has New York to equal it?"

"Murray Hill," said a pretty young thing in pink.

=The chief of the Minneapolis fire department grows facetious and enlivens the otherwise dull pages of this report with a flash of wit when, in enumerating the causes of fires, he mentions "looking for leak in gas pipe with a lighted lamp and found it—one."

=A Nebraska historian who is writing a history Nance County, in order to get to the beginning, has gone back to the creation of the world. He cannot decide, however, whether it was God who created it or "whether it was evolved by self-operating forces inherent in matter." He is coming down to Nance County by easy stages by way of Adam and the flood.

=The students of a Western theological seminary are reported to have discussed the question whether, in the case of a prayer having been read from a printed slip on a formal occasion, and there having been a typographical error entirely reversing the meaning of a passage, the petition was received by Providence as uttered or as originally written. The debaters spent a whole evening over the point, and then had a tie vote.

="You horrid thing!" said Miss Jaggs to Miss Minnie Jaggs, when they had sought their room; "you actually ate that biscuit after Uncle Horace had reached it to you on his own fork!" "Well what of it?" "What of it? I should say what of of it! The idea of touching anything that anyboby else's fork has been in!" "It doesn't make any difference in Uncle Horace's case." "Why not?" "Because he never puts his fork in his mouth."

="I should hate to be a lawyer," remarked the grocer, " and be obliged to argue contrary to my belief." Then he prcceeded to inform a customer that the bottle of cotton-seed oil he held in his hand was the pure unadulterated juice of the olive. "I should hate to be a grocer," said the lawyer, "and have to soil my hands with molasses, kerosene and all sorts of vile stuff." He was seen a few moments later shaking hands with a murderer, a burglar and a drunkard.

=Dr. R. von Lendenfeld, who has been studying the sponges of the Australian shores for the Linnean Society of New South Wales, has succeeded in discovering the nervous system of these low animals, which has hitherto escaped observation. This discovery is of very great scientific interest, inasmuch as it proves the much disputed animal nature of sponges beyond all doubt. The nervous system consists of small miodermal, spindle-shaped cells, similar to those ectodermal elements which perform the functions of sensitive cells in jellyfish and higher animals. Ganglia cells have also been detected similar to those in higher animals.

=At a cost of nine million of francs Paris has built a new post-office and placed it on the site of the old one, erected in the year 1757. The French capital has since stretched westward, but still the Rue Jean Jacques Rousseau remains the center of the great city on the Seine. An improvement has been adopted which might be imitated here. A large room is set apart for persons who may desire to write letters. It is furnished with maps, guide books, dictionaries, and directories, and has a supply of pens, inks, and blotting paper. An attendant supplies stationery at cost price, and there is a small charge of two cents.

=M. John Lemoinne, in the *Journal des Debats*, laments the want of enterprise manifested by the French industrial classes, as compared with those of Great Britain and the United States. He says that Frenchman are not only over cautious, but such great lovers of routine that it is impossible to get them out of a path they have once fairly entered into. As an example of this stolidity of character, he instances the condition of the advertising trade in France. Although Frenchmen know that Englishmen and Americans have made large fortunes by continuous and persevering advertising, few Frenchmen are inclined to seek fortune in that way. As for newspaper advertising in France, it is still in its infancy, and is likely to remain so, for Frenchmen will not see that the presence of numerous advertisements in the same paper in no way prevents the public from giving attention to an individual advertisement.

=An Arizona sporting man was recently inveigled into a church fair, and induced to try his hand at the wheel of fortune. In half an hour he had all the money in the bank and a mortgage on the church. He very considerately gave them back the mortgage, but in the future the church will choose its victims with more care.

=The Rev. A. P. Harper, D. D., figures out a steady decrease in the population of China. He says the present number of inhabitants cannot exceed 300,000,000. Chief among the causes of diminution is opium. He believes that the population of India will soon exceed that of China, the latter ceasing to be the most populous country on the globe.

=India-ink markings on the skin are often the source of much annoyance in after life to those who have been tattooed in youth. The editor of the *Medical Press* states that nothing but the knife or an active escharotic will produce a satisfactory result. He holds that the cutis must be destroyed. Dr. Neale, of London, however, claims that he has secured good results with sodii ethylas, first suggested by Dr. B. W. Richardson. He cites the case of a gentleman who in his younger days had a palm tree tattooed on his arm and an elaborate bracelet on his wrist, who has quite lost all the dark matter, etc., the skin being left in many places quite natural, although there is still more or less of a scar, but this diminishes month by month.

=A man breathes about eighteen times a minute and uses 3,000 cubic feet of air per hour.

=It is estimated that 2,000 persons a year, mostly prisoners, take their own lives in Russia.

=The bee has long been a type of the industrious worker, but there are few people who know how much labor the sweet hoard of the hive represents. Each head of clover contains about sixty distinct flower tubes, each of which contains a portion of sugar not exceeding the five-hundredth part of a grain. Some patient aparian enthusiast who has watched their movements concludes that the proboscis of the bee must therefore be inserted into 500 clover tubes before one grain of sugar can be obtained. There are 7,000 grains in a pound, and as houey contains three-fourths of its weight of dry sugar, each pound of honey represents 2,500,000 clover tubes sucked by bees.

=The French medical journals have recently been discussing the relation of teeth to the brain, and their conclusions are of considerable importance to brain-workers. It seems to have been clearly established that excessive and prolonged mental labor causes the teeth to decay by consuming the phosphates which would otherwise nourish the dental structures.

—All communications for this magazine should be addressed to HALL'S JOURNAL OF HEALTH, 75 & 77 Barclay Street, New York.

